# E-cadherin expression in the tumor microenvironment of advanced epidermal growth factor receptor-mutant lung adenocarcinoma and the association with prognosis

**DOI:** 10.1186/s12885-023-10980-6

**Published:** 2023-06-20

**Authors:** Yu-Ping Chang, Gong-Kai Huang, Yung-Che Chen, Kuo-Tung Huang, Yu-Mu Chen, Chiung-Yu Lin, Chao-Cheng Huang, Meng-Chih Lin, Chin-Chou Wang

**Affiliations:** 1 Division of Pulmonary and Critical Care Medicine, Department of Internal Medicine, Kaohsiung Chang Gung Memorial Hospital, Chang Gung University College of Medicine, Kaohsiung, Taiwan; 2 Department of Pathology, Kaohsiung Chang Gung Memorial Hospital, Chang Gung University College of Medicine, Kaohsiung, Taiwan; 3 Department of Laboratory Medicine, Kaohsiung Chang Gung Memorial Hospital, Chang Gung University College of Medicine, Kaohsiung, Taiwan; 4 Department of Respiratory Therapy, Kaohsiung Chang Gung Memorial Hospital, Chang Gung University College of Medicine, Kaohsiung, Taiwan; 5 Biobank and Tissue Bank, Department of Pathology, Kaohsiung Chang Gung Memorial Hospital, Chang Gung University College of Medicine, Kaohsiung, Taiwan; 6 Department of Respiratory Care, Chang Gung University of Science and Technology, Chiayi, Taiwan

**Keywords:** Adenocarcinoma, E-cadherin, Epidermal growth factor receptor (EGFR), Lung cancer, Programmed death-ligand 1 (PD-L1), Tumor-infiltrating lymphocytes, Vimentin

## Abstract

**Background:**

The expression of programmed death-ligand 1 (PD-L1), tumor-infiltrating lymphocytes (TILs), E-cadherin, and vimentin in lung cancer tumor microenvironment is known to impact patient survival or response to therapy. The expression of these biomarkers may also differ between primary lung tumors and brain metastatic tumors. In this study, we investigated the interaction between these biomarkers in lung tumors with or without concomitant brain metastasis and the interaction with paired brain metastatic tumors.

**Methods:**

The study included 48 patients with stage IV epidermal growth factor receptor (EGFR)-mutant lung adenocarcinoma. Sixteen of the forty-eight patients were diagnosed with brain metastasis, while the remaining thirty-two were not. All sixteen patients with brain metastasis had brain tumors. The expression of PD-L1, TILs (CD8^+^ T lymphocytes and FOXP3^+^ regulatory T lymphocytes), E-cadherin, and vimentin were evaluated using immunohistochemical (IHC) staining.

**Results:**

Patients with brain metastasis exhibited a higher frequency of exon 19 deletion and uncommon EGFR mutations, a higher lung tumor vimentin score, worse progression-free survival (PFS), and overall survival (OS) than patients without brain metastasis. IHC staining showed no difference between paired lung and brain tumors. Patients with low PD-L1 expression had better PFS and OS. After multivariate analysis, higher body mass index, the presence of brain metastasis, bone metastasis, and uncommon EGFR mutations were correlated with worse PFS, while the presence of brain metastasis and high lung tumor E-cadherin score was associated with worse OS.

**Conclusions:**

In patients with stage IV EGFR-mutant lung adenocarcinoma, high E-cadherin expression in the lung tumor might be associated with worse OS. Vimentin expression in the lung tumor was positively related to the risk of brain metastasis.

## Background

Lung cancer is a leading cause of cancer-related death worldwide despite advances in treatment [1]. In East Asia, approximately half of lung adenocarcinoma (ADC) patients have epidermal growth factor receptor (EGFR) mutations, and tyrosine kinase inhibitor (TKI) therapy is the standard treatment for advanced EGFR-mutant lung ADC [2]. Brain metastasis (BM) is more common in EGFR-mutant non-small cell lung cancer (NSCLC) than in wild-type NSCLC [3], and the prognosis is poor if patients develop BM [4, 5]. Advances in immune checkpoint inhibitors (ICIs) targeting the programmed death-1 (PD-1)/programmed death-ligand 1 (PD-L1) pathway, such as pembrolizumab, improved survival compared with platinum-based chemotherapy in advanced NSCLC patients with PD-L1 expression of at least 50% and without EGFR mutation or anaplastic lymphoma kinase gene translocation [6]. A study led by Akbay and colleagues revealed that activation of the EGFR pathway resulted in PD-L1 upregulation along with an immunosuppressive tumor microenvironment (TME) characterized by a lower CD8^+^/CD4^+^ and CD8^+^/FOXP3^+^ tumor-infiltrating lymphocytes (TILs) ratio in a mouse model, and a blockade with PD-1 antibody improved survival [7]. A meta-analysis of nivolumab (CheckMate 057), pembrolizumab (KEYNOTE-010), and atezolizumab (POPLAR) confirmed that ICIs as a second-line treatment prolonged the overall survival (OS) over docetaxel in wild-type EGFR but not in EGFR-mutant advanced NSCLC patients [8]. In the TME, interaction between PD-L1, CD8^+^ TILs, tumor-infiltrating FOXP3^+^ regulatory T lymphocytes (Tregs) was also reported [9]. Among them, CD8^+^ TILs were associated with favorable outcomes and played an important role in cell-mediated antitumor response and were associated with favorable outcomes [10, 11], whereas tumor-infiltrating FOXP3^+^ Tregs were thought to have inhibitory effects on antitumor immunity and correlated to a worse prognosis in lung cancer patients [12]. In several malignancies, the CD8^+^/ FOXP3^+^ TILs ratio is also associated with improved patient survival [13]. TILs of brain metastatic tumors also have a potential prognostic value [5], and NSCLC brain metastatic tumors have a higher mutational burden and fewer T-cell clones compared with primary lung tumors [14]. Despite the presence of TILs, PD-L1 expression was found to be associated with epithelial-mesenchymal transition (EMT) in lung ADC [15]. EMT is a process in which carcinoma cells metastasize and invade organs and may contribute to drug resistance [16].

We investigated the interaction between PD-L1, TILs represented by CD8^+^ T lymphocytes and FOXP3^+^ Tregs, and EMT represented by E-cadherin and vimentin expression. In this study, we evaluated the expression of these immune biomarkers in lung tumors with or without concomitant BM and with paired brain metastatic tumors.

## Methods

### Participants

**Fig. 1:**
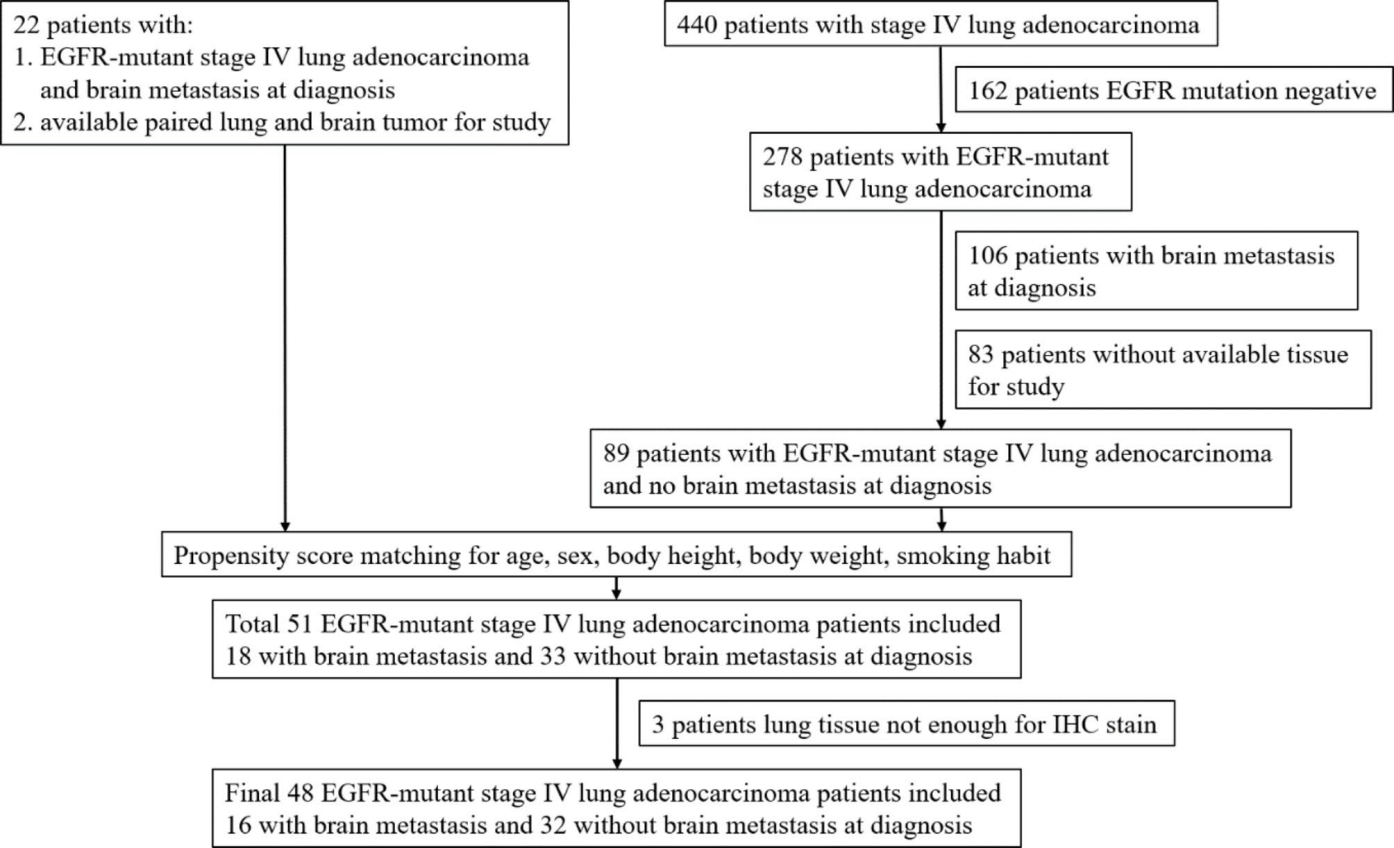
Inclusion and exclusion criteria flow chart

Twenty-two over 20 years old stage IV EGFR-mutant lung ADC patients with BM at diagnosis having paired lung and brain tumors were selected for the study between 2015/01/01 to 2019/12/31 from the patient database of the Department of Pathology, Kaohsiung Chang Gung Memorial Hospital, Taiwan. We also retrospectively reviewed medical records of patients over 20 years old diagnosed with stage IV lung ADC, between 2015/01/01 and 2019/12/31 at Kaohsiung Chang Gung Memorial Hospital, Taiwan. Eighty-nine stage IV EGFR-mutant lung ADC patients without BM at diagnosis were selected for propensity score matching (PSM). Eighteen patients with BM and 33 patients without BM at diagnosis were selected after PSM, but the lung tissues of three patients were not sufficient for immunohistochemical (IHC) staining. Therefore, the analysis included sixteen patients with BM and 32 without BM at diagnosis. The inclusion and exclusion criteria are described in the flow chart presented in Fig. 1.

Lung ADC was staged according to the AJCC 8th edition criteria [17]. The routine workup for lung cancer staging includes chest computed tomography, brain magnetic resonance imaging, and bone scans. Pleural effusion cytology studies and positron emission tomography were performed if needed. Progression-free survival (PFS) was defined as the period from the first day of treatment to documented disease progression or death before disease progression. Overall survival (OS) was defined as the period from the first day of treatment to death. Disease progression was determined following response evaluation criteria in solid tumors (RECIST) version 1.1 [18]. Performance status (PS) was defined based on the eastern cooperative oncology group (ECOG) criteria [19]. The follow-up time was defined as the first day of treatment to the last follow-up date and was 948.0 (603.8–1360.3) days in the median. EGFR mutation analysis was performed by real-time polymerase chain reaction using the therascreen**®** EGFR RGQ PCR kit (Qiagen, Hilden, Germany) or cobas**®** EGFR Mutation Test v2 kit (Roche Molecular systems, CA, USA) with formalin-fixed and paraffin-embedded tissue according to per manufacturers’ protocol. This study was approved by the Institutional Review Board of Chang Gung Memorial Hospital (IRB: 202000369B0D001 and 202200538B0).

### Immunohistochemical staining of tissues

The hematoxylin and eosin (H&E)-stained sections of lung and brain tumors were assessed. Lung tumors were obtained only at the time of diagnosis, while brain tumors were obtained at the time of diagnosis or during the treatment course. A total of 48 formalin-fixed, paraffin-embedded lung tissue samples and sixteen brain tissues were collected and submitted for an IHC study. Using monoclonal antibodies against CD8 (rabbit, clone SP16, Thermo Fisher Scientific, Fremont, USA), FOXP3 (mouse, clone 150D, BioLegend, San Diego, USA), vimentin (rabbit, clone SP20, Thermo Fisher Scientific, Fremont, USA), and E-cadherin (mouse, clone GM016, Genemed Biotechnologies, South San Francisco, USA), an automated IHC analysis was performed by the following systems: BenchMark Ultra System (Ventana Medical Systems, Mannheim, Germany) for CD8; Leica BOND-III automated immunostainer (Leica Biosystems, Wetzlar, Germany) for FOXP3; and i6000™ Infinity System (BioGenex, CA, USA) for vimentin and E-cadherin. In addition, the anti-PD-L1 antibody clone 22C3 (Agilent/Dako, Santa Clara, USA), and a prototype IHC assay with a Dako Autostainer Link 48 platform (Agilent Technologies, Santa Clara, USA) were also used to determine the PD-L1 tumor proportion score. Slides were evaluated by two pathologists (GKH and CCH), who were blind to the clinicopathological data.

**Fig. 2:**
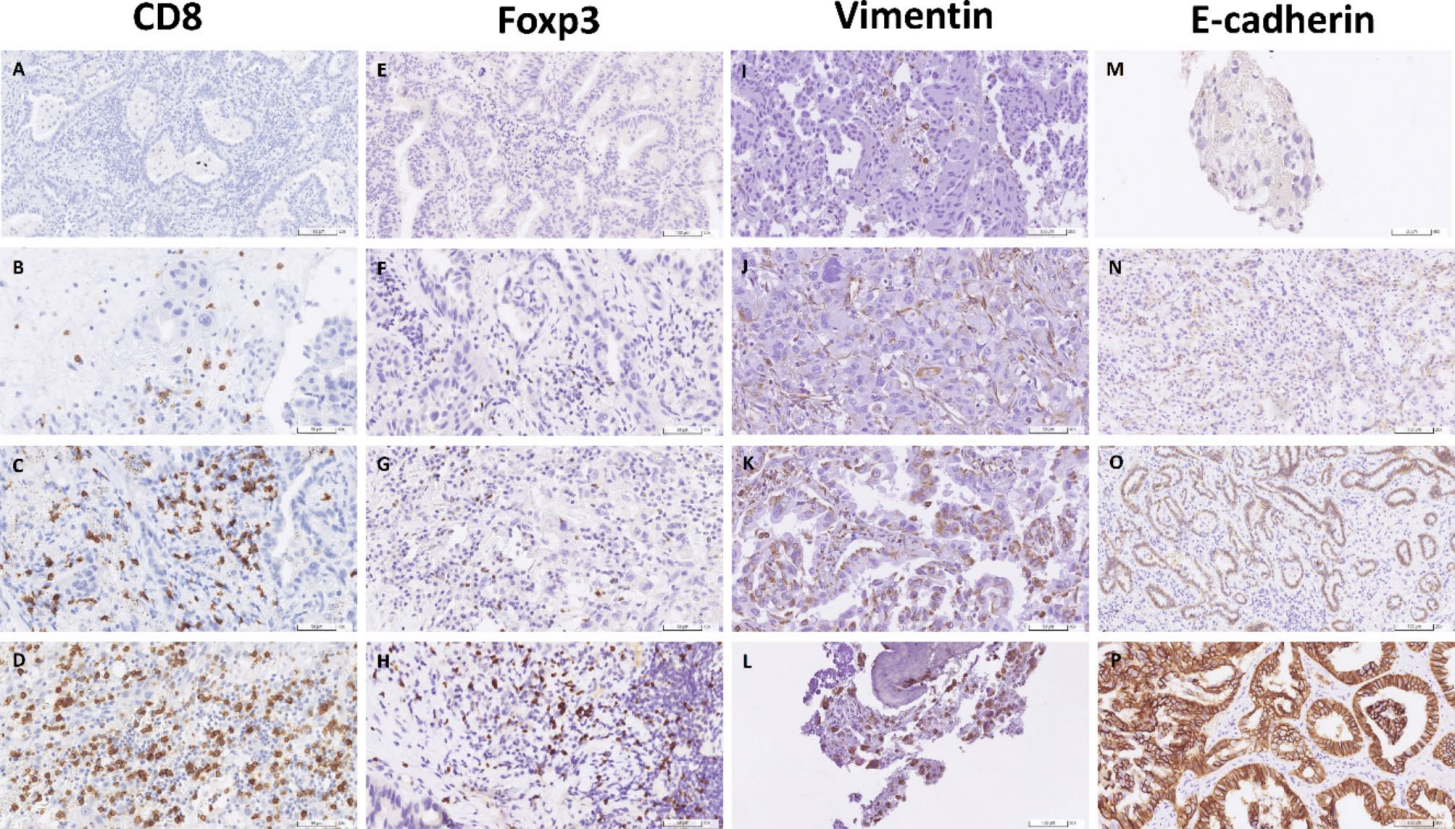
Representative images of CD8, FOXP3, E-cadherin, and vimentin immunohistochemical staining. **A** to **D**, CD8^+^ cell count at the hot spot = 0, < 100/HPF, 100–200/HPF, and > 200/HPF. **E** to **H**, FOXP3^+^ cell count at the hot spot = 0, < 50/HPF, 50–100/HPF, and > 100/HPF. **I** to **L**, vimentin expression by tumor cells, interpreted as (0), (1+, 40%), (2+, 30%), and (3+, 90%). **M** to **P**, E-cadherin expression by tumor cells, interpreted as (0), (1+, 5%), (2+, 90%), and (3+, 100%). HPF: high-power field

**Fig. 3:**
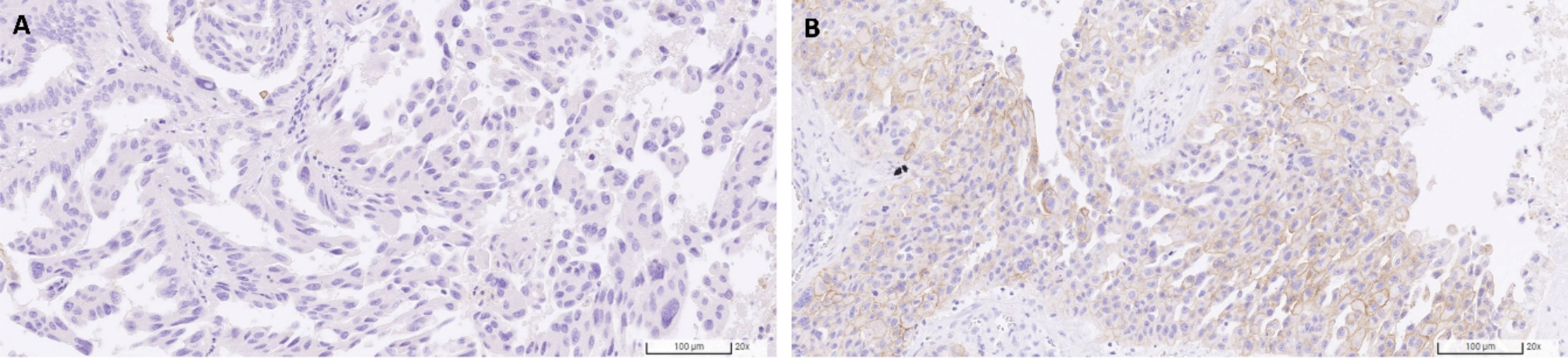
Representative images of PD-L1 immunohistochemical staining. **A**, TPS < 1%. **B**, TPS = 20%. TPS: Tumor proportion score

The tissue sections were analysed by light microscopy (Olympus BX43F, Tokyo, Japan) for the degree of infiltration by CD8^+^ and FOXP3^+^ T lymphocytes. The number of CD8^+^ and FOXP3^+^ cells were counted in a selected 0.238mm^2^ field area hotspot under 400× magnification. In the case of E-cadherin and vimentin, the staining intensity was graded as per the membranous expression: 0 equals to no expression; 1 equals to fragmented membranous and/or weak to moderate expression; 2 equals to fragmented strong or fully membranous moderate expression; and 3 equals to fully membranous strong expression. The percentage of immunoreactive positive tumor cells was graded as: 0 (no positive tumor cells), 1 (less than 10% positive tumor cells), 2 (10–50% positive tumor cells), and 3 (more than 50% positive tumor cells) [20]. An expression score was defined as the product of the percentage of immunoreactive positive tumor cells graded and the staining intensity. The score could be graded as 0, 1, 2, 3, 4, 6, or 9. The representative images of CD8, FOXP3, E-cadherin, and vimentin stains are shown in Fig. 2 and of PD-L1 expression are shown in Fig. 3.

### Statistical analysis

Continuous variables were presented as median with interquartile range and compared using the non-parametric Mann–Whitney U-test, whereas the categorical variables were presented as frequency with percentage and compared by the Chi-square test. PFS and OS were analyzed using Kaplan–Meier curves and log-rank testing. A Cox proportional hazards regression model was used to evaluate independent factors influencing survival outcomes, and all covariates with a *p*-value < 0.1 were included for analysis. Statistical analyses were performed using SPSS version 21.0 (IBM Corp., Armonk, New York, USA). Statistical significance was set at a *p*-value of < 0.05.

PSM was conducted to balance the differences in clinical characteristics between patients with BM at diagnosis and those without BM. Propensity scores were calculated using logistic regression analysis and covariates included sex, body height, body weight, age, and smoking habits. Patients with BM at diagnosis were matched 1:2 to patients without BM using nearest-neighbor matching with a caliper at 0.2. Standardized differences for a covariate were set at < 10.0%. PSM was performed using NCSS version 11.0.5 (NCSS LLC., Kaysville, UT, USA).

## Results

### Patient characteristics

A total of sixteen patients with BM and 32 without BM at diagnosis were included; the clinical characteristics and IHC staining results of lung tumors are listed in Table 1.

**Table 1:** Clinical characteristics and immunohistochemical staining results of lung tumors of 48 patients

	With brain metastasis (n = 16)	No brain metastasis (n = 32)	*p*-value
Sex			0.759
Male	7 (43.8%)	12 (37.5%)	
Female	9 (56.3%)	20 (62.5%)	
Age (years)	56.0 (52.0–65.5)	60.8 (53.3–66.0)	0.477
BMI (kg/m^2^)	23.9 (21.8–25.2)	23.3 (21.8–25.2)	0.710
Smoking habits			1.000
Never	13 (81.3%)	27 (84.4%)	
Former/current	3 (18.8%)	5 (15.6%)	
ECOG PS			0.090
0	7 (43.8%)	6 (18.8%)	
≥ 1	9 (56.3%)	26 (81.3%)	
Comorbidities			
Hypertension Diabetes mellitus	4 (25.0) 3 (18.8%)	15 (46.9) 6 (18.8%)	0.213 1.000
COPD	1 (6.3%)	3 (9.4%)	1.000
Hyperlipidemia	2 (12.5%)	4 (12.5%)	1.000
Chronic kidney disease	3 (18.8%)	9 (28.1%)	0.725
Extrapulmonary metastasis			
Liver	4 (25.0%)	4 (12.5%)	0.413
Bone	7 (43.8%)	16 (50.0%)	0.765
EGFR mutation			**0.018**
Exon 19 deletion	9 (56.3%)	11 (34.4%)	
L858R mutation	3 (18.8%)	19 (59.4%)	
Others	4 (25.0%)	2 (6.3%)	
First-line TKI			0.347
Gefitinib	4 (25.0%)	3 (9.4%)	
Erlotinib	3 (18.8%)	8 (25.0%)	
Afatinib	9 (56.3%)	21 (65.6%)	
PFS (median, days)	358.0	830.0	**0.018**
OS (median, days)	930.0	1671.0	**0.012**
Lung tumor IHC stain			
PD-L1			0.894
< 1%	9 (56.3%)	17 (53.1%)	
1–50%	4 (25.0%)	10 (31.3%)	
> 50%	3 (18.8%)	5 (15.6%)	
PD-L1 (median)	1.0 (0-12.5)	1.0 (1.0–35.0)	0.503
CD8^+^ cell counts/HPF	55.5 (8.5–97.5)	52.5 (2.5-107.5)	0.895
FOXP3^+^ cell counts/HPF	37.5 (12.5–76.3)	21.0 (2.8–88.8)	0.375
E-cadherin score	6.0 (6.0–9.0)	6.0 (6.0–9.0)	0.696
Vimentin score	1.5 (1.0-3.5)	1.0 (0.0-1.8)	**0.034**

BMI: body mass index; COPD: chronic obstructive pulmonary disease; ECOG: Eastern Cooperative Oncology Group; EGFR: epidermal growth factor receptor; HPF: high-power field; IHC: immunohistochemical; OS: overall survival; PFS: progression-free survival; PS: performance status; TKI: tyrosine kinase inhibitor

There was no difference in sex, age, BMI, smoking habits, ECOG PS, comorbidities, liver or bone metastasis, or first-line TKI categories between these two groups. Patients with BM at diagnosis had more frequent exon 19 deletions and uncommon EGFR mutations, less frequent L858R mutations, and worse PFS and OS than patients without BM at diagnosis. IHC stain of lung tumors from these two groups showed no difference in PD-L1 expression, CD8^+^ TILs, tumor-infiltrating FOXP3^+^ Tregs, or E-cadherin score. Patients with BM at diagnosis had considerably higher lung tumor vimentin scores than those without. Four of 32 patients without BM at diagnosis developed BM later, and the analysis of lung tumors of these four patients displayed a trend of higher vimentin scores than those 28 patients who did not develop BM during follow-up [median: 1.5 (1.0–3.5) vs. 0.5 (0.0–1.0); *p*-value = 0.072].

### IHC staining results of paired lung and brain tumors from patients with brain metastasis at diagnosis

IHC staining results of PD-L1, CD8, FOXP3, E-cadherin, and vimentin from paired lung and brain tumors in patients with BM at diagnosis are listed in Table 2.

**Table 2:** Immunohistochemical staining results of sixteen paired lung and brain tumors

	Lung tumor (n = 16)	Brain tumor (n = 16)	*p*-value
PD-L1			0.856
<1%	9 (56.3%)	9 (56.3%)	
1–50%	4 (25.0%)	5 (31.3%)	
>50%	3 (18.8%)	2 (12.5%)	
PD-L1 (median)	1.0 (0.0-12.5)	1.0 (1.0–16.3)	0.590
CD8^+^ cell counts/HPF	55.5 (8.5–97.5)	108.5 (44.8–167.5)	0.073
FOXP3^+^ cell counts/HPF	37.5 (12.5–76.3)	18.0 (12.3–56.0)	0.381
E-cadherin score	6.0 (6.0–9.0)	9.0 (6.0–9.0)	0.110
Vimentin score	1.5 (1.0-3.5)	1.5 (0.0–2.0)	0.305

HPF: high-power field

There was no significant difference observed in the expression of PD-L1, CD8^+^ TILs, tumor-infiltrating FOXP3^+^ Tregs, E-cadherin, and vimentin score between lung and brain tumors. Compared to brain tumors with low PD-L1 expression (< 1%), lung tumors with low PD-L1 expression exhibited significantly lower CD8^+^ TILs [median: 29.0 (4.0–89.0) vs. 125.0 (61.5–230.0); *p*-value = 0.011].

### PD-L1 expression and association with prognosis

The clinical characteristics and IHC stain results of lung tumors stratified by PD-L1 expression are listed in Table 3. There was no difference in sex, age, BMI, smoking habits, ECOG PS, EGFR mutation type, first-line TKI categories, or proportion of brain, liver, and bone metastasis between these two groups. IHC stain of CD8^+^ TILs, tumor-infiltrating FOXP3^+^ Tregs, E-cadherin score, and vimentin score was not different between these two groups. Low PD-L1 expression patients had significantly longer PFS and OS than patients with high PD-L1 expression.

**Table 3:** Forty-eight patients were categorized by lung tumor PD-L1 expression

Parameters	PD-L1 < 1% (n = 26)	PD-L1 ≥ 1% (n = 22)	*p*-value
Sex			0.771
Male	11	8	
Female	15	14	
Age (years)	60.7 (54.0–66.0)	57.6 (48.7–67.7)	0.535
BMI (kg/m^2^)	23.3 (20.9–25.4)	23.6 (22.2–25.0)	0.772
Smoking habits			0.260
Never	20 (76.9%)	20 (90.9%)	
Former/current	6 (23.1%)	2 (9.1%)	
ECOG PS			0.532
0	6 (23.1%)	7 (31.8%)	
≥ 1	20 (76.9%)	15 (68.2%)	
Extrapulmonary metastasis			
Brain	9 (34.6%)	7 (31.8%)	1.000
Liver	5 (19.2%)	3 (13.6%)	0.710
Bone	9 (34.6%)	14 (63.6%)	0.081
EGFR mutation			0.516
Exon 19 deletion	12 (46.2%)	8 (36.4%)	
L858R mutation	12 (46.2%)	10 (45.5%)	
Others	2 (7.7%)	4 (18.2%)	
First-line TKI			0.109
Gefitinib	3 (11.5%)	4 (18.2%)	
Erlotinib	9 (34.6%)	2 (9.1%)	
Afatinib	14 (53.8%)	16 (72.7%)	
PFS (median, days)	972.0	500.0	**0.013**
OS (median, days)	2077.0	1222.0	**0.044**
IHC stain			
CD8^+^ cell counts/HPF	53.0 (6.0–92.5)	55.0 (11.3–145.3)	0.755
FOXP3^+^ cell counts/HPF	16.5 (0.0–64.3)	45.0 (13.3–89.8)	0.054
E-cadherin score	6.0 (6.0–6.0)	6.0 (6.0–9.0)	0.137
Vimentin score	1.0 (0.0–2.0)	1.0 (0.8–2.0)	0.469

BMI: body mass index; ECOG: eastern cooperative oncology group; EGFR: epidermal growth factor receptor; HPF: high-power field; IHC: immunohistochemical; OS: overall survival; PFS: progression-free survival; PS: performance status; TKI: tyrosine kinase inhibitor

### Independent factors affecting PFS and OS

Independent factors associated with PFS are listed in Table 4. Univariate analysis revealed that younger age, the presence of brain and bone metastasis, uncommon EGFR mutations, and high PD-L1 expression (≥ 1%), were all associated with worse PFS. Higher BMI, the presence of brain and bone metastasis, and uncommon EGFR mutations were found to be factors associated with poorer PFS in multivariate analysis.

**Table 4:** Cox regression analysis of factors related to 1st line progression-free survival

Parameters	Univariate analysis	Multivariate analysis
	HR (95% CI) *p*-value	HR (95% CI) *p*-value
Age (years)	0.953 (0.913–0.994) **0.024**	0.140
Sex (male vs. female)	0.822 (0.386–1.750) 0.611	
BMI (kg/m^2^)	1.092 (0.991–1.204) 0.076	1.165 (1.040–1.305) **0.008**
Never vs. Former/current	1.172 (0.468–2.937) 0.735	
ECOG PS (≥ 1 vs. 0)	1.422 (0.582–3.476) 0.440	
Extrapulmonary metastasis		
Brain	2.405 (1.136–5.088) **0.022**	3.994 (1.695–9.410) **0.002**
Liver	1.829 (0.745–4.489) 0.187	
Bone	2.435 (1.144–5.182) **0.021**	3.188 (1.465–6.939) **0.003**
EGFR mutation		
Exon 19 deletion, L858R	0.248 (0.091–0.679) **0.007**	0.229 (0.080–0.654) **0.006**
Others	1	
1st -line TKI		
Afatinib	0.612 (0.298–1.259) 0.182	
Erlotinib, Gefitinib	1	
IHC stain		
PD-L1 (≥ 1% vs. < 1%)	2.446 (1.177–5.082) **0.017**	0.090
CD8^+^ cell counts/HPF	1.001 (0.997–1.004) 0.737	
FOXP3^+^ cell counts/HPF	0.997 (0.990–1.004) 0.384	
E-cadherin score		
= 9	2.249 (0.958–5.281) 0.063	0.536
< 9	1	
Vimentin score		
≥ 2	1.108 (0.518–2.373) 0.791	
< 2	1	

BMI: body mass index; ECOG: Eastern Cooperative Oncology Group; EGFR: epidermal growth factor receptor; HPF: high-power field; IHC: immunohistochemical; PS: performance status; TKI: tyrosine kinase inhibitor

**Fig. 4:**
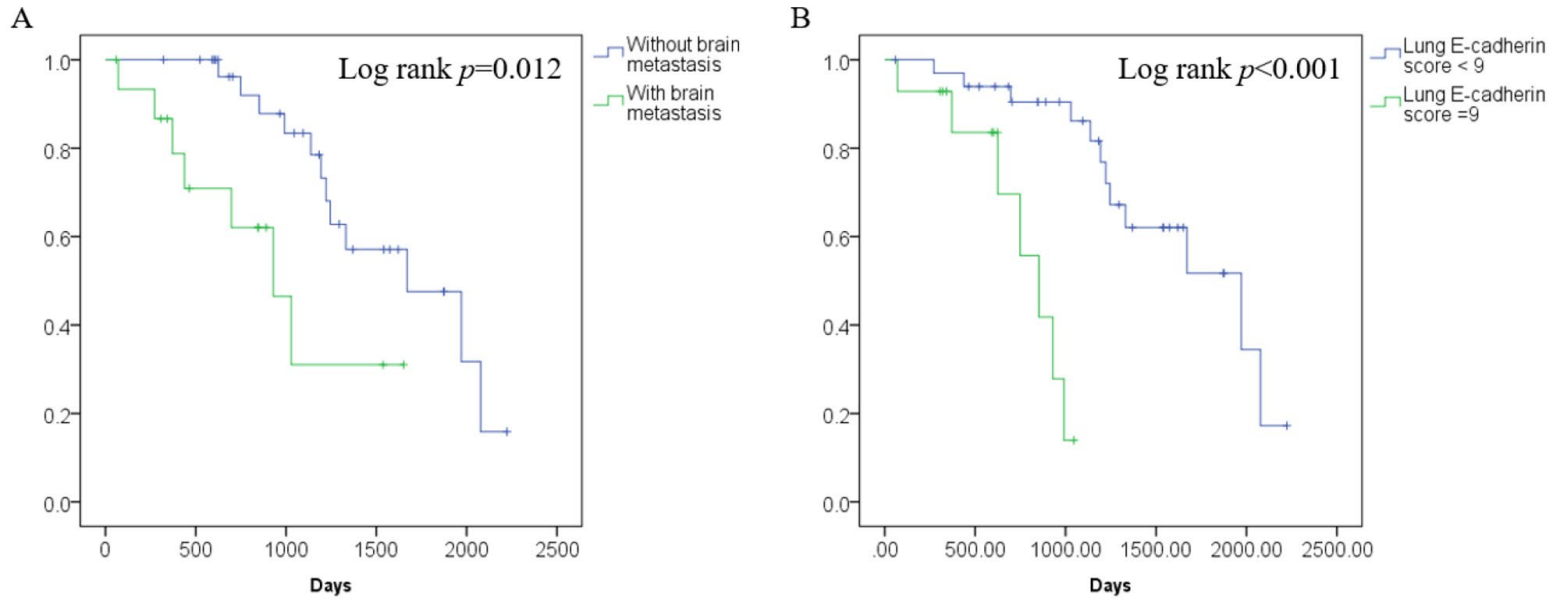
Independent factors associated with overall survival. **A**, brain metastasis. **B**, lung E-cadherin score

Independent factors associated with OS are listed in Table 5. Both univariate and multivariate analysis revealed the presence of BM, and high E-cadherin scores were associated with worse OS. Kaplan–Meier curves of OS regarding BM and E-cadherin scores are shown in Fig. 4.

**Table 5:** Cox regression analysis of factors related to overall survival

Parameters	Univariate analysis	Multivariate analysis
	HR (95% CI) *p*-value	HR (95% CI) *p*-value
Age (years)	0.955 (0.902–1.012) 0.118	
Sex (male vs. female)	1.270 (0.480–3.359) 0.630	
BMI (kg/m^2^)	1.094 (0.953–1.255) 0.201	
Never vs. Former/current	3.003 (0.662–13.611) 0.154	
ECOG PS (≥ 1 vs. 0)	0.954 (0.270–3.368) 0.942	
Extrapulmonary metastasis		
Brain	3.401 (1.237–9.345) **0.018**	3.704 (1.334–10.285) **0.012**
Liver	1.541 (0.343–6.931) 0.573	
Bone	1.993 (0.770–5.157) 0.155	
EGFR mutation		
Exon 19 deletion, L858R	0.209 (0.040–1.090) 0.063	0.268
Others	1	
`1st line TKI		
Afatinib	1.285 (0.460–3.592) 0.632	
Erlotinib, Gefitinib	1	
IHC stain		
PD-L1 (≥ 1% vs. < 1%)	2.657 (0.989–7.133) 0.053	0.408
CD8	0.998 (0.992–1.005) 0.585	
FOXP3	0.995 (0.984–1.006) 0.360	
E-cadherin score		
= 9	9.487 (2.631–34.207) **0.001**	10.281 (2.795–37.815) **<0.001**
< 9	1	
Vimentin score		
≥ 2	0.709 (0.253–1.987) 0.513	
< 2	1	

BMI: body mass index; ECOG: Eastern Cooperative Oncology Group; EGFR: epidermal growth factor receptor; HPF: high-power field; IHC: immunohistochemical; PS: performance status; TKI: tyrosine kinase inhibitor

## Discussion

### PD-L1 expression in stage IV EGFR-mutant lung adenocarcinoma and correlation with tumor microenvironment

Previous studies by Santaniello and colleagues about the relationship between EGFR mutation and PD-L1 expression showed conflicting results; however, they concluded that it might be due to different PD-L1 evaluation methods and interpretations [9]. Activation of the EGFR pathway has been shown to induce PD-L1 expression in mouse models and NSCLC cell lines [7], and the pathway may involve yes-associated protein (YAP) [21]. In our hospital, a larger cohort showed lower PD-L1 expression in EGFR-mutant NSCLC patients than in wild-type NSCLC patients [22].

In our cohort, there was no correlation of CD8^+^ TILs, tumor-infiltration FOXP3^+^ Tregs, E-cadherin, or vimentin scores with PD-L1 expression in lung TME. Though a tendency for high PD-L1 expression in lung tumors related to higher tumor-infiltrating FOXP3^+^ Treg counts was observed. Activation of EGFR pathways in NSCLC was associated with decrease in CD8^+^ TILs [23, 24], but an increase in tumor-infiltrating FOXP3^+^ Tregs [25]. This difference may be due to the small population size and different patient cohorts.

PD-L1 expression in our cohort showed no correlation with E-cadherin and vimentin scores but, EMT was reported to be related to PD-L1 overexpression in lung adenocarcinoma, especially in the EGFR-mutant subgroup [15]. Asgarova et al. demonstrated that cytokine-induced EMT in lung cancer cell lines could induce PD-L1 upregulation and vimentin expression correlated with PD-L1 expression in NSCLC patients [26]. Thus, the correlation between PD-L1 expression and EMT still needs further investigation.

Although among the sixteen paired lung and brain tumors, there were no differences in IHC stain results, as listed in Table 2. Higher CD8^+^ TILs in brain tumors compared with lung tumors were observed, and low PD-L1 expression (< 1%) brain tumors had significantly higher CD8^+^ TILs than low PD-L1 expression lung tumors [median: 125.0 (61.5–230.0) vs. 29.0 (4.0–89.0); *p* = 0.011]. Some previous studies suggested that in NSCLC patients, brain metastatic tumors have less PD-L1 expression [27] and fewer TILs [14, 27] compared with primary tumors, but others did not [28, 29]. Notably, brain metastatic tumor PD-L1 expression was found to be strongly correlated with primary lung tumor in lung adenocarcinoma patients and no significant change was found to be affected by chemotherapy or steroid therapy. However, the majority of patients in this study were wild-type patients [29]. The different results may be due to different patient populations. In EGFR-mutant NSCLC patients, rebiopsy of the lung tumor after TKI resistance showed increased PD-L1 expression, with decreased CD8^+^ and FOXP3^+^ TIL densities [30]. This could also partially explain why our results differ from other reports because eight of sixteen patients’ brain tissues were obtained after first-line TKI treatment.

### Epithelial-mesenchymal transition manifested by vimentin expression and risk of brain or other distant metastases

EMT plays a crucial role in lung cancer progression and metastasis. It is characterized by decreased E-cadherin expression and vimentin overexpression [31], and also participates in the mechanism of TKI resistance in EGFR-mutant lung adenocarcinoma [32]. In our cohort, lung tumors from patients with initial BM showed significantly higher vimentin expression than lung tumors from patients without initial BM. But this is not the case with bone or liver metastasis. Vimentin expression in NSCLC has been linked to future metastasis [33], and pathologic stage and N status [34]. Jeevan and colleagues demonstrated that the EMT/MET pathway is crucial for BM from lung adenocarcinoma [35]. Our study validated the role of EMT in lung cancer BM.

### Factors associated with patient outcome

In our cohort, higher BMI and the presence of brain and bone metastasis were independently associated with unfavorable PFS, while the common EGFR mutation was independently associated with better PFS. The association of BMI and lung cancer prognosis differs between studies and race, sex, smoking habits, and lung cancer subtypes [36]. In our study, higher BMI became significantly associated with poorer PFS by multivariate analysis, this may be due to different patient population since our study focused on patients with EGFR-mutant lung adenocarcinoma and relatively small population size. BM [4, 5] and bone metastasis [37] also adversely affect patient survival, as observed in our patient cohort. Uncommon EGFR mutations showed varied responses to TKIs [38]. NSCLC patients harboring common EGFR mutations had a better response to TKIs and a better prognosis than rare EGFR mutations [39], as occurred in our cohort.

Although patients with higher PD-L1 expression had a worse outcome in our cohort, it was not statistically significant according to multivariate analysis with PFS (*p* = 0.090) and OS (*p* = 0.408). Indeed, advanced EGFR-mutant lung adenocarcinoma patients with higher PD-L1 expression had worse PFS [40, 41], OS, and a lower frequency of secondary T790M mutation [41].

The presence of BM and high E-cadherin expression were both independent factors associated with worse OS in our study. As summarized in a meta-analysis [42], low E-cadherin expression was associated with poor prognosis in NSCLC patients as well as a group of NSCLC patients treated with chemoradiotherapy [43]. The presence of aberrant E-cadherin expression or loss of E-cadherin expression was associated with worse outcomes in other cancers such as melanoma [44], gastric cancer [45], and colorectal cancer [46]. Although the patient cohort is different, our study showed the opposite result that a high E-cadherin score correlates with poor OS. Indeed, there was evidence that cancers with high E-cadherin expression showed aggressive behavior and an unfavorable outcome, such as, in a subgroup of human brain glioblastoma, E-cadherin expression was associated with aggressive behavior and could be blocked by shRNA in a cell line study [47]. Despite E-cadherin expression in IHC stain, cleaved E-cadherin fragments (soluble E-cadherin) may have an oncogenic effect, increase tumor cell motility and survival, and play a role in EGFR and Wnt/β-catenin pathway signaling [48]. Elevated serum soluble E-cadherin levels were found to be associated with disease invasiveness and a poor outcome in several cancers [48]. Additionally, erlotinib and gefitinib could reduce E-cadherin expression in human papillomavirus 16-positive and -negative cell lines [49]. Taken together, it is unclear whether high E-cadherin expression leads to high soluble E-cadherin after TKI treatment. In other cancers, the correlation of serum soluble E-cadherin and E-cadherin expression in IHC stain was studied, which was not compatible with bladder cancer [50] and hepatocellular carcinoma [51]. Also, studies on breast, gastric, and colorectal cancers have stated that serum soluble E-cadherin level is inversely correlated with E-cadherin expression in tissues [52], but whether this correlation applies to EGFR-mutant lung adenocarcinoma needs further investigation.

### Limitations

There are a few limitations to our study. Our study may have been limited due to the small population size. Additionally, we obtained eight of the sixteen patients brain tissue samples after TKI treatment, which could affect inflammation status. For example, Isomoto et al. demonstrated that TKI treatment altered the TME by expressing PD-L1, CD8^+^ TILs, or tumor-infiltrating FOXP3^+^ Tregs [30]. The inhomogeneous spatial distribution of PD-L1 [53] and TILs [54] in NSCLC patients tumors could also affect our interpretation since our specimens were from a partial tumor biopsy.

## Conclusions

Our study revealed the possible role of E-cadherin and vimentin expression in EGFR-mutant lung adenocarcinoma. In patients with stage IV EGFR-mutant lung adenocarcinoma, high E-cadherin expression in the lung tumor might be associated with worse OS, and vimentin expression in the lung tumor was positively related to the risk of brain metastasis. E-cadherin expression might be a useful biomarker in evaluating prognosis and vimentin expression in evaluating risk of brain metastasis. Further studies are recommended to clarify the role of these biomarkers in the pathogenesis of EGFR-mutant lung adenocarcinoma.

## Data Availability

The datasets used and/or analyzed during the current study are available from the corresponding author upon reasonable request.

## List of abbreviations

ADC: Adenocarcinoma
BM: Brain metastasis
ECOG: Eastern Cooperative Oncology Group
EGFR: Epidermal growth factor receptor
EMT: Epithelial-mesenchymal transition
ICIs: Immune checkpoint inhibitors
IHC: Immunohistochemical
NSCLC: Non-small cell lung cancer
OS: Overall survival
RECIST: Response evaluation criteria in solid tumors
PD-1: Programmed death-1
PD-L1: Programmed death-ligand 1
PFS: Progression-free survival
PS: Performance status
PSM: Propensity score matching
TILs: Tumor-infiltrating lymphocytes
TKI: Tyrosine kinase inhibitor
TME: Tumor microenvironment
Tregs: Regulatory T lymphocytes
